# Do human tumor-associated viruses play a role in the development of synovial sarcoma?

**DOI:** 10.1186/s13569-015-0027-x

**Published:** 2015-04-15

**Authors:** Ulrich Lenze, Florian Pohlig, Heinrich Mühlhofer, Florian Lenze, Andreas Toepfer, Hans Rechl, Rainer Burgkart, Rüdiger von Eisenhart-Rothe, Melanie Straub

**Affiliations:** Department for Orthopedics and Orthopedic Sports Medicine, Klinikum rechts der Isar, Technical University, 81675 Munich, Germany; Institute of Pathology, Klinikum rechts der Isar, Technical University Munich, Munich, Germany

**Keywords:** Virus, Soft tissue sarcoma, Synovial sarcoma, EBV, HPV, HHV-8

## Abstract

**Background:**

To date, the pathomechanism of soft tissue sarcomas such as synovial sarcoma remains unclear whereas even a viral etiology was suspected. Aim of this study was to analyze whether EBV, HHV-8 or HPV play a role in the development of synovial sarcomas.

**Findings:**

In total 41 synovial sarcomas were included in this retrospective study. For detection of EBV 1/2 and HHV-8, resection specimens were analyzed with regard to virus-specific sequences using a SingleStep PCR. HPV analysis was carried out by an HPV-specific multiplex-PCR and subsequent array-hybridization for HPV-typing. No virus-specific DNA of EBV, HHV-8 or HPV was detected.

**Conclusion:**

An involvement of these viruses in the etiology of synovial sarcoma was not detected but further studies are needed with different virus types and sarcoma entities.

## Findings

Synovial sarcoma (SS) is a malignant soft tissue tumor, which is mostly seen during the 3rd decade of life, but approximately 30% of cases occur in patients younger than 20 years [1,2]. The etiology of pediatric soft tissue sarcomas such as synovial sarcoma remains unclear whereas even a viral cause was suspected [3]. Beside the Hepatitis B and C viruses, especially the Epstein Barr Virus (EBV), the Human Herpes Virus-8 (HHV-8) and the Human Papilloma Virus (HPV) are considered to be responsible for the majority of infection-attributable cancer cases worldwide [4]. The latter 3 viruses belong to a group of different DNA and RNA viruses, which play a role in the oncogenesis of various carcinomas and even sarcomas. EBV, for example seems to contribute to the pathogenesis of leiomyosarcomas in immunodeficient patients, especially in children [5]. However, an involvement of these viruses in the pathogenesis of other soft tissue tumors such as synovial sarcoma has not been proven to our knowledge. Therefore, the aim of this study was to analyze whether EBV, HHV-8 or HPV play a role in the development of this entity.

## Material and methods

**Figure 1:**
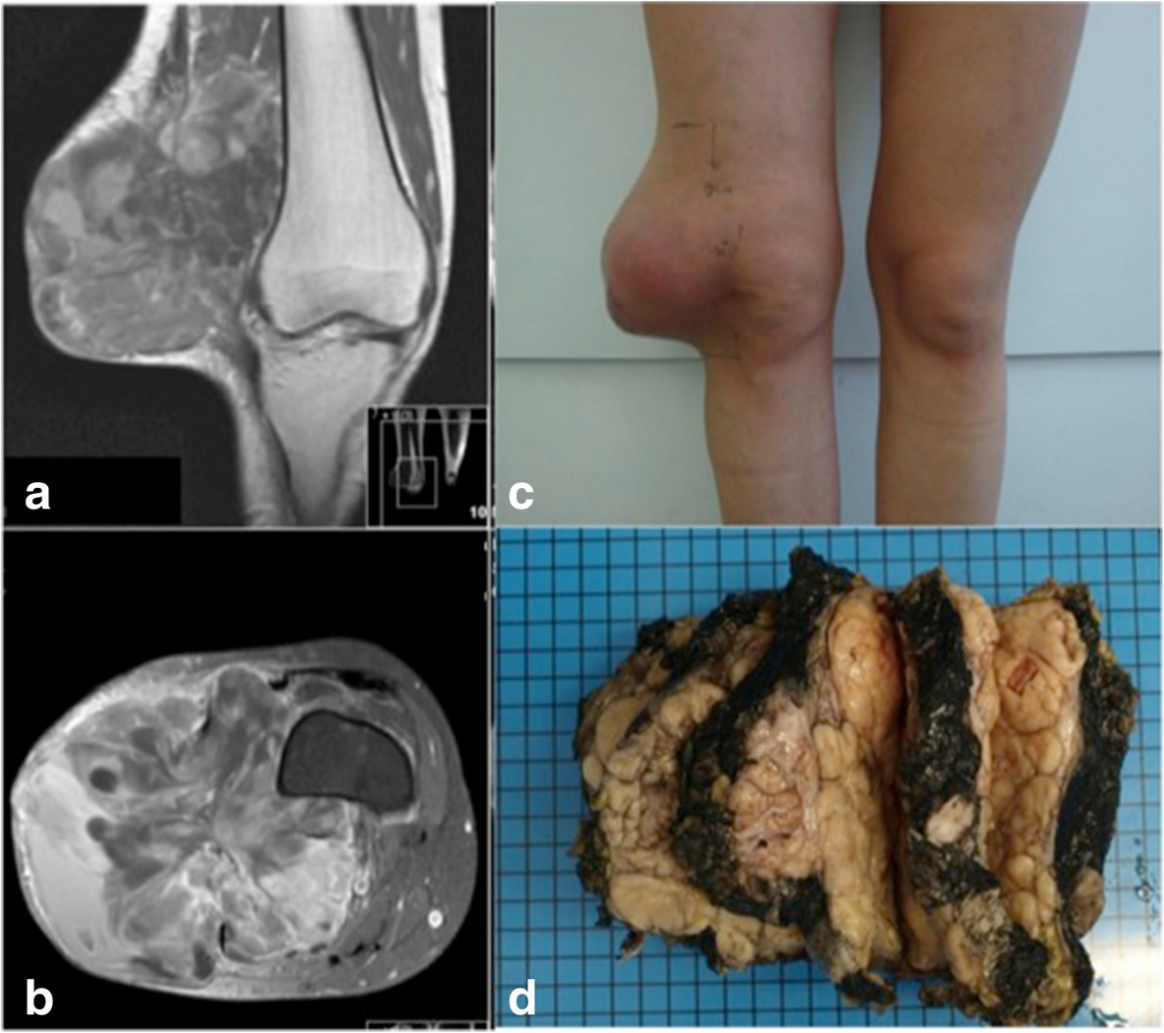
Clinical and radiological appearance of synovial sarcoma: Female patient (30 y. old) with synovial sarcoma of the right knee: **a)** coronar T1-weighted MRI, **b)** axial T1-weighted and fat saturated MRI with contrast agent, **c)** clinical picture bevor limb - salvage surgery, **d)** cross-sections through the resected tumour.

In total 41 synovial sarcomas were included in this retrospective study (Figure 1). Tumor resection specimens were formalin-fixed and paraffin-embedded. All samples - either primary tumor or metastasis/recurrence - were derived from different patients (Table 1). Diagnosis was established by histology and immunohistology. Diagnosis of synovial sarcoma was confirmed by the presence of a SYT-SSX fusion transcript caused by t(X;18) by RT-PCR. For detection of EBV 1/2 and HHV-8, virus-specific sequences were analyzed using a SingleStep PCR. HPV analysis was carried out by an HPV-specific multiplex-PCR and subsequent array-hybridization for HPV-typing. Additionally, an in-situ-hybridization analysis for detection of EBV was performed. The study was approved by the institutional review board of the Technical University of Munich.

**Table 1:** **Summary of patients and tumor characteristics**

**Case**	**Age**	**Gender**	**Localisation**	**Tumor**	**Specimen**	**Morphology**	**Grading**	**Molecular data**	**Virus analysis**
		**m (male) f (female)**		**p (primary) m (metastasis) r (recurrence)**	**n (naive) y (pretreatment)**	**m (monophasic) b (biphasic) d (dedifferentiated)**	**G1 (well differentiated) G2 (moderately differentiated) G3 (poorly differentiated) n.a. (not available)**		**+ (positive) - (negative)**
1	40	f	groin	p	n	b	G3	SSX1	-
2	50	f	thigh	p	n	b	G3	SSX1	-
3	31	m	lungs	m	n	m	G3	SSX2	-
4	47	f	knee	p	n	m	G3	SSX1	-
5	29	m	thigh	r	n	b	G3	SSX1	-
6	6	f	forearm	p	n	m	G2	SSX1	-
7	36	f	thigh	p	n	m	G2	SSX1	-
8	44	m	foot	p	y	m	n.a	SSX1	-
9	52	f	foot	p	n	b	G3	SSX1	-
10	26	m	lower leg	p	n	b	G3	SSX1	-
11	64	f	shoulder	p	n	b	G2	SSX1	-
12	56	f	thigh	p	n	m	G3	SSX1	-
13	70	f	thigh	p	n	b	G3	SSX1	-
14	35	m	upper arm	p	y	m	G3	SSX2	-
15	42	m	forearm	p	n	b	G3	SSX2	-
16	49	f	lungs	m	n	m	G3	SSX2	-
17	53	m	lungs	p	n	m	G3	SSX1	-
18	34	f	trunk	p	n	m	G2	SSX2	-
19	21	f	shoulder	p	n	b	G2	SSX1	-
20	54	f	thigh	p	y	m	G3	SSX1	-
21	16	m	knee	p	n	b	G2	SSX1	-
22	85	f	forearm	p	n	m	G2	SSX1	-
23	21	f	knee	p	n	m	G2	SSX1	-
24	24	m	buttock	r	n	m	G3	SSX1	-
25	46	m	thigh	p	y	m	G3	SSX2	-
26	37	f	lungs	m	n	b	G2	SSX1	-
27	41	m	lungs	m	n	b	G3	SSX1	-
28	30	f	knee	p	n	m	G3	SSX2	-
29	74	f	buttock	p	n	m	G3	SSX2	-
30	17	f	thigh	p	n	m	G3	SSX1	-
31	30	f	thigh	p	n	m	G2	SSX1	-
32	20	f	trunk	p	n	b	G2	SSX2	-
33	35	m	trunk	m	n	m	G2	SSX2	-
34	43	m	lungs	m	n	b	G3	SSX1	-
35	35	m	thigh	p	y	m	G3	SSX1	-
36	51	m	trunk	r	n	m	G2	SSX1	-
37	48	w	knee	r	y	m	G2	SSX2	-
38	65	m	lower leg	p	n	m	G2	SSX2	-
39	48	m	thigh	p	n	b	G3	SSX1	-
40	30	m	head	p	n	m	G2	SSX2	-
41	75	w	thigh	p	n	m	G2	SSX2	-

## Results

An adequate amount of tumor tissue was available in all patients in order to perform and evaluate the analysis. No virus-specific DNA of EBV, HHV-8 or HPV were detected in synovial sarcomas (n = 41).

## Discussion

As in most soft tissue sarcomas, the etiology of SS has not been completely uncovered. Although the involvement of a fusion gene resulting from a specific chromosomal translocation t(X;18) (p11;q11) was disclosed, which is detected in more than 95%, SS remains an entity of uncertain origin [6,7]. Therefore, treatment options of this high-grade sarcoma are reduced, whereas patients often develop distant metastases leading to a 10-years survival of <50% [8].

It is estimated, that up to 18% of cancer diseases are associated with viral infections [4]. The human virus HPV contributes to 5,5% of all cancers, EBV to 1% and HHV-8 together with the human immunodeficiency virus (HIV) to 0,9% of all cancer cases [4]. However, although significantly high antibody titers were found in patients with soft tissue sarcomas e.g. for EBV, there is no clear evidence that virus infections constitute a major risk factor in the development of soft tissue sarcomas [5,9]. Nevertheless, since certain childhood cancer diseases seem to have seasonal variations, some authors suspected a viral etiology [10,11].

We therefore investigated in this study, whether virus-specific DNA of EBV, HHV-8 or HPV is detectable in tumor resection specimens of patients with synovial sarcoma. We conclude that, although these viruses contribute to the oncogenesis of a considerable number of malignant tumors, an involvement in the pathogenesis of synovial sarcoma was not detected. Further investigations with different sarcoma entities and virus types are to be conducted.

## Abbreviations

SS: Synovial Sarcoma
EBV: Epstein Barr Virus
HHV-8: Human Herpes Virus-8
HPV: Human Papilloma Virus
DNA: Deoxyribonucleic acid
RNA: Ribonucleic acid
PCR: Polymerase Chain Reaction
RT-PCR: Reverse Transcription Polymerase Chain Reaction
HIV: Human Immunodeficiency Virus

## References

[1] Okcu MF, Munsell M, Treuner J, Mattke A, Pappo A, Cain A (2003). Synovial sarcoma of childhood and adolescence: a multicenter, multivariate analysis of outcome. J Clin Oncol.

[2] Wolden SL, Alektiar KM (2010). Sarcomas across the age spectrum. Semin Radiat Oncol.

[3] Fletcher CD, Unni KK, Mertens F (2002). World Health Organization Classification of Tumours. Pathology and genetics of tumours of soft tissue and bone.

[4] Parkin DM (2006). The global health burden of infection-associated cancers in the year 2002. Int J Cancer J Int Cancer.

[5] Kebudi R, Bilgic B, Gorgun O, Ayan I, Demiryont M (2003). Is the Epstein Barr virus implicated in Ewing sarcoma?. Med Pediatr Oncol.

[6] Nagayama S, Katagiri T, Tsunoda T, Hosaka T, Nakashima Y, Araki N (2002). Genome-wide analysis of gene expression in synovial sarcomas using a cDNA microarray. Cancer Res.

[7] Clark J, Rocques PJ, Crew AJ, Gill S, Shipley J, Chan AM (1994). Identification of novel genes, SYT and SSX, involved in the t(X;18)(p11.2;q11.2) translocation found in human synovial sarcoma. Nat Genet.

[8] Bergh P, Meis-Kindblom JM, Gherlinzoni F, Berlin O, Bacchini P, Bertoni F (1999). Synovial sarcoma: identification of low and high risk groups. Cancer.

[9] Weiss SW, Goldblum JR, Enzinger FM (2001). Enzinger and Weiss’s soft tissue tumors.

[10] van Laar M, Kinsey SE, Picton SV, Feltbower RG (2013). First description of seasonality of birth and diagnosis amongst teenagers and young adults with cancer aged 15–24 years in England, 1996–2005. BMC Cancer.

[11] Ross JA, Severson RK, Swensen AR, Pollock BH, Gurney JG, Robison LL (1999). Seasonal variations in the diagnosis of childhood cancer in the United States. Br J Cancer.

